# Optimization Strategies and Efficiency Prediction for Silicon Solar Cells with Hybrid Route of PERC and SHJ Passivation Contact

**DOI:** 10.1002/advs.202411965

**Published:** 2025-02-24

**Authors:** Zixiao Zhou, Qian Kang, Zhaoqing Sun, Yongcai He, Jingjie Li, Lu Wu, Chang Sun, Chaowei Xue, Minghao Qu, Xiaoqing Chen, Zilong Zheng, Bo Wang, Hui Yan, Xixiang Xu, Yongzhe Zhang

**Affiliations:** ^1^ College of Materials Science and Engineering Key Laboratory of New Functional Materials of Ministry of Education Beijing University of Technology Beijing 100124 China; ^2^ LONGi Green Energy Technology Co., Ltd Xi'an 710016 China; ^3^ LONGi Central R&D Institute Xi'an 712000 China; ^4^ School of Information Science and Technology Key Laboratory Optoelectronics Technology of Ministry of Education Beijing University of Technology Beijing 100124 China

**Keywords:** FELA, hybrid solar cell, passivation contact, simulation

## Abstract

PERC solar cell technology, which emerged in the 1980s, has garnered a substantial portion of the PV market over the past decade. However, the main factors limiting their further efficiency advancements and wider commercialization lie in metal contact recombination and the passivation properties of the functional layers. Despite heterojunction cells demonstrating remarkable efficiency, challenges persist in terms of cost reduction and stability enhancement. This study introduces a novel hybrid solar cell architecture that integrates a diffusion‐free front surface field with a high‐quality heterojunction passivation contact. Through rigorous simulation analysis, it is revealed that the hybrid design surpasses conventional PERC in several key aspects: diminished front‐surface recombination losses, enhanced rear‐contact characteristics, and reduced grid shading. By strategically optimizing the front passivation and adopting single‐side wet etching techniques, a PCE of 24.17% and *V*_OC_ of 716 mV is successfully achieved on a full‐size commercial czochralski silicon wafer (274.15 cm^2^). Additionally, both experimental *EQE* tests and simulations delve into the composition of *J*_SC_ gain during the optimization process. This comprehensive investigation not only offers an in‐depth assessment of hybrid solar cell performance, but also outlines promising avenues for future optimization aimed at pushing theoretical efficiency limits further and enhancing suitability for large‐scale production.

## 1 Introduction

As crystalline silicon (*c*‐Si) solar cell technology matures, its levelized cost of energy (LCOE) continues to decline, securing a significant market share in renewable energy‐based electricity consumption.^[^ ^1^, ^2^, ^3^, ^4^, ^5^ ^]^ To further challenge traditional technology and reduce industrialization cost, innovative approaches for *c*‐Si cells are imperative. Building upon the foundational generations of photovoltaic technology, including aluminum back surface field (Al‐BSF)^[^ ^6^ ^]^ and passivation emitter rear contact (PERC),^[^ ^7^ ^]^ advancements like passivation emitter rear totally‐diffused (PERT)^[^ ^7^, ^8^ ^]^ and passivation emitter rear locally‐diffused (PERL)^[^ ^7^, ^8^, ^9^ ^]^ cells have demonstrated improved contact properties, notably low defect‐assisted Shockley‐Read‐Hall (SRH) recombination rate and contact resistivity (*ρ*_c_), through rear‐side functional‐impurity doping. However, these PERC+ processes introduce complexity and additional efficiency losses due to Auger recombination and free carrier absorption (FCA).^[^ ^10^ ^]^ Although PERC accounted for almost 80% of the total shipment in 2023, according to various predictions of photovoltaic institutions,^[^ ^11^, ^12^, ^13^ ^]^ its market share will be reduced to ≈20% or less by the end of 2024.

To address these limitations, effective selective passivation strategies have emerged in high efficiency *c*‐Si technologies, notably tunneling oxide passivation contact (TOPCon)^[^ ^14^, ^15^, ^16^ ^]^ and silicon heterojunction (SHJ),^[^ ^17^, ^18^, ^19^, ^20^ ^]^ leveraging ultra‐thin passivation layers such as multiple‐pinhole silicon oxide (SiO_x_)^[^ ^21^ ^]^ or intrinsic hydrogenated amorphous silicon (i‐*a*‐Si:H).^[^ ^17^, ^18^ ^]^ The SHJ approach involves depositing a stack of intrinsic and doped hydrogenated amorphous silicon (*a*‐Si:H) layer^[^ ^22^ ^]^ on the *c*‐Si surface via plasma‐enhanced chemical vapor deposition (PECVD), effectively passivating dangling bonds and reducing interface recombination. Furthermore, the low‐velocity deposition buffer layer in i‐*a*‐Si:H, rich in hydrogen and featuring a large microstructure factor (R*), inhibits epitaxial and nanotwins growth.^[^ ^17^, ^18^, ^19^, ^22^ ^]^

While excellent passivation properties are crucial, they often compromise transport and optical performance, leading to fill factor (*FF*) and short current density (*J*_SC_) losses.^[^ ^23^, ^24^, ^25^ ^]^ In view of the low conductivity of doped amorphous silicon, the sputtering of transparent conductive oxide (TCO) is employed to enhance lateral transport of majority carriers.^[^ ^26^, ^27^ ^]^ However, the TCO/*a*‐Si:H stack introduces parasitic absorption, energy band mismatch, other issues like high indium consumption and toxic gas emissions.^[^ ^28^, ^29^ ^]^ Substituting amorphous silicon with hydrogenated nanocrystalline silicon oxide (*nc*‐SiO_x_:H)^[^ ^30^, ^31^, ^32^ ^]^ can suppress parasitic absorption and improve conductivity, but oxygen incorporation significantly impacts device stability. Consequently, researchers have explored wide‐bandgap, high transmittance materials for novel selective contacts, notably doping‐free asymmetric heterojunction (DASH)^[^ ^33^, ^34^ ^]^ and transparent passivating contact (TPC),^[^ ^35^ ^]^ achieving remarkable photoelectric conversion efficiency (PCE) of 23.5% and 23.99%, respectively.

Herein, we propose a hybrid structure that balances passivation, contact, and optical performance. The free energy and loss analysis is employed to compare the device performance of PERC and hybrid solar cells. Leveraging the advantage of P‐diffusion n+ structure without front surface field (FSF), we propose a SiO_x_ passivation route to replace SiO_x_ (n+) and assess leakage in the n‐p contact region through dark *I–V* measurements. Additionally, we adopt a single etching method to preserve front layer passivation. Based on experimental tests and simulation, we analyze *J*_SC_ losses in these routes and predict optimized metallization methods. Finally, the optimized champion device achieves a PEC of 24.17% and an open‐circuit voltage (*V*_OC_) of 716 mV. And the possible optimization routes in the future are presented at the end of this article. While the efficiency lags behind state‐of‐the‐art *c*‐Si cells like TOPCon and SHJ, we present a novel concept and methodology for exploring high‐performance, mass‐producible hybrid solar cells.

## 2 Results and Discussion

### 2.1 Simulation Analysis and Characterization for Hybrid Solar Cells

**Figure 1:**
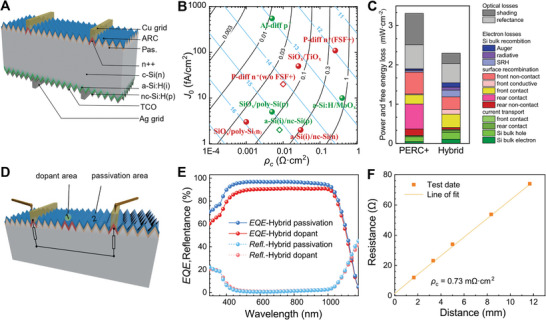
A) Schematic diagram of the hybrid solar cell. B) parameters of different selective transport contacts. C) FELA analysis of hybrid and PERC+ solar cells and device performance of PERC and hybrid solar cells. D) Schematic diagram of tests method and location. E) *EQE* and reflectance curves of passivation and dopant area shown in points of inset (b). F) TLM measurement results of front side.

**Figure** **1**A illustrates our hybrid structure based on an n‐type *c*‐Si wafer of ≈170 µm we use to demonstrate the device details in this work. The local n+ dopant structure ensures the selective transport of electron and the full front surface is subsequently modified by passivation/ARC layers deposited at high temperature. On the other side, the 30‐µm‐thick *a*‐Si:H(i)/*nc*‐Si:H(p) stack grows on the fresh texture to passivate surface dangling bonds of *c*‐Si. For metallization, plating copper and screen‐printing silver are applied as electrodes for carrier collection. The schematic diagram of external quantum efficiency (*EQE*) and transmission line method (TLM)^[^ ^36^ ^]^ tests is shown in Figure 1D, where the green dot 1 is the reserved dopant layer to characterize its parasitic absorption loss. The theoretical limiting efficiency of silicon solar cells is usually predicted by the selectivity of their passivation and contact properties.^[^ ^37^, ^38^, ^39^ ^]^ The correlation of selectivity and electrical parameters is shown in Figure 1B, where the red and green dots represent electron and hole selective contact structure, respectively. To assess the potential *η*_max_ of silicon‐based solar cells and provide guidance for experimental endeavors, the following calculation equations have been derived by fitting to the curve of maximum power points:^[^ ^37^, ^38^, ^39^ ^]^

(1) $$\eta_{max}={\left({\left(2.452\;S_{10}-4.240\right)}^{-19.52}+{\left(29.21\right)}^{-19.52}\right)}^{-\frac{1}{19.52}}\;\%$$

(2) $$S_{10,e\&h}=log_{10}\;\left(\frac{V_{th}}{J_{0,e\&h}\;\rho_{c,e\&h}}\right)$$

where the *S*_10_ is selectivity coefficient, logarithmically related to the saturation current density (*J*_0_) and *ρ*_c_. And *V*_th_ is the thermal voltage. In Brendel's model, the optical losses and detailed structure of solar cell is not considered. The front junction (FJ) and back junction (BJ) silicon solar cells are demonstrated to have different FSF design rules. As depicted in Figure 1B, the phosphorus‐diffused (P‐diff) emitter, equipped with a FSF and positioned on the front side of the PERC solar cell, exhibits inferior properties owing to structural constraints. In this study, we have applied the P‐diff contact, devoid of a FSF, to the front‐side non‐contact area of our designed structure. Therefore, the front sheet resistant of non‐contact region will no longer be limited to meet the lateral transport of major carrier, which provides a significant opportunity for improvement of front passivation property. Benefit to the bulk transport of BJ solar cell, the selectivity of P‐diff contact without FSF is modified to a better level (highlighted by the red diamond in Figure 1B). On the other hand, the design of full area passivation contact on the back is necessary for BJ solar cell because it requires efficient selective transport for carrier in emitter. Therefore, the *a*‐Si(i)/*nc*‐Si(p) stack stands out as an optimal choice, attributed to its exceptional passivation qualities and electron properties (highlighted by the green diamond in Figure 1B). And the comprehensive full‐area coverage of the TCO layer enhances carrier collection efficiency.

The 2D simulation tool Quokka 3 was used to perform the simulated properties of PERC and hybrid solar cells. The input parameters were derived from experimental measurement and literature extraction, as detailed in Table S1 (Supporting Information).^[^ ^37^, ^38^, ^39^, ^40^ ^]^ In Figure 1C, the free energy and power loss of hybrid and traditional PERC solar cells are compared in terms of optical, recombination, and transport in our simulation. The primary advantage of hybrid solar cells in terms of optical losses stems from the reduced metal shading associated with plated copper.^[^ ^41^, ^42^ ^]^ For comparison, Figure S1A,B (Supporting Information) depict the surface morphologies of silver grids produced by screen‐printing and copper grids formed by plating, respectively. It is difficult to preserve the morphology of the silver grid as original screen‐printing after annealing. Figure S1C,D (Supporting Information) exhibit the cross‐section views of copper grid with dense Ni/Cu/Ni structure, the finger morphology (height and width) can be well controlled by plating current and time. As discussed previously, non‐essential limit for front sheet resistance make hybrid solar cell displays less front non‐contact loss caused by surface recombination. Another performance improvement comes from the reduction of rear non‐contact and contact recombination loss, which derives from the hydrogen passivation of defect amorphous silicon and avoidance of metal/semiconductor direct contact with isolation of TCO.

The *EQE* and reflectance spectra tested with micrometer diameter spot of the dopant and passivation/ARC the region are shown in Figure 1E, and the test positions correspond to two dots in Figure 1D. The loss of *EQE* curves in the short wavelength comes from reflectance of front texture and absorption of SiN_x_ film, where the refractive index (*n*) and extinction coefficient (*k*) of SiN_x_ is provided in Figure S2 (Supporting Information). The full wavelength reduction of red curve is duo to the parasitic absorption of dopant layer. The resistance with different pitches between test grids is shown in Figure 1F, and the results are fitted by TLM method to calculate parameters such as contact resistivity (0.73 mΩ·cm^2^) and sheet resistance.

### 2.2 Optimization Strategy of Passivation and Wet Etching Routes

**Figure 2:**
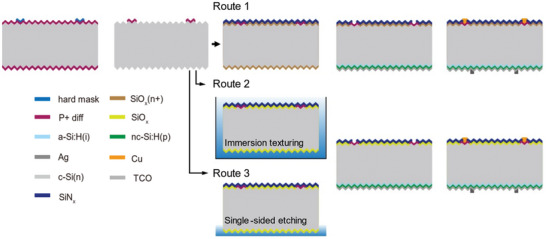
The fabrication illustration with different processes of hybrid solar cells optimizing route.

**Figure 3:**
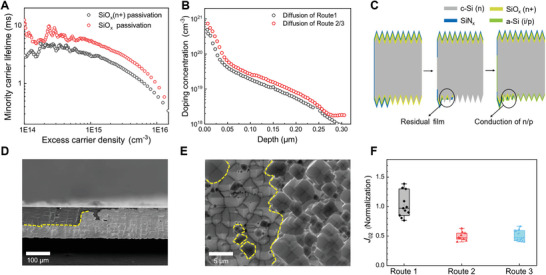
A) The lifetime of sample with SiO_x_ (n+) and SiO_x_ passivation; B) the *ECV* curves of sample with different routes; C) schematically model of back side and edge of device through serious wet process; the D) cross‐section and E) surface view of edge undergoing pickling etching and alkali texturing; F) the comparison of normalization *J*_02_ parameters with continuous optimization.

The schematics of different fabrication routes of hybrid solar cell are shown in **Figure** **2**. The electron‐selective contact area is protected by hard mask such as film deposition or printing ink in step 1, the localized graphical design is achieved by laser or photolith. Next, unmasked area is etched by alkali to form new random pyramid, and hard mask is removed. The deposition of a phosphorus‐doped SiO_x_ (n+)/SiN_x_ stack fulfills a dual function in route 1, serving both as a front passivation layer and exhibiting anti‐reflection properties.^[^ ^43^ ^]^ The subtle diffusion of phosphorous generates a weak field that aligns with the polarity of the fixed charge in SiO_x_, enhancing passivation uniformity and yielding a front surface with reduced sheet resistance. However, phosphorus diffusion causes extra Auger recombination in silicon bulk and the residual region would increase the risk of leakage at the rear p‐n junction. In route 2, we employ un‐dopant ultra‐thin SiO_x_ (3 nm) as the front passivation layer and match the corresponding SiN_x_ process, whose selectivity is located in Figure 1B as P‐diff n+ without FSF. **Figure** **3**A illustrates the trend of minority carrier density over time for SiO_x_ (n+)/SiN_x_ and SiO_x_/SiN_x_ structures. The black curve represents the passivation achieved via Route 1, exhibiting a lifetime of 1.6 ms, while the red curve corresponds to the passivation obtained through Routes 2 and 3, with a lifetime of 2.3 ms. The electrochemical capacitance‐voltage profiler (*ECV*) measurement curves with different dopant concentrations of selectivity region are shown in Figure 3B. It is demonstrated that an n+ layer with high doping concentration of surface ensures low contact resistance with metal grid.^[^ ^44^ ^]^

In the preparation of hybrid route, adequate secondary texturing to ensure the surface clean for rear side is very important to get high‐quality passivation performance with the deposition of intrinsic amorphous silicon. During the annealing process of SiN_x_, a large number of pinholes are formed with the escape of hydrogen. The immersion texturing method in routes 1 and 2 causes etchant penetration through pinholes to destroy passivation properties. The single‐side etching is an effective approach to protect the passivation on the front side in route 3, which increases the open voltage and current density. Notably, the silicon nitride deposited in tubular equipment creates additional coverage on the cross‐section and back edges of the wafer. And the residual silicon nitride acts as a mask layer to block the secondary texturing of edge and backside, leading to the formation of residual n+ layers and inverted pyramids, as shown in the yellow dotted lines in Figure 3D,E, respectively. As previously discussed in route 1, the SiO_x_ (n+) film is also protected by residual SiN_x_ film in backside and eventually exposed at the edge of the inverted pyramid after secondary texturing. With the next step of p‐type amorphous silicon deposition, leakage points form at the edges of these inverted pyramids, and the schematic representation of the process is shown in Figure 3C. The normalized *J*_02_ values of different routes are extracted by dark‐state *IV* curves and compared in Figure 3F. At low voltages, the ideality factor of approaches two and the parameter *J*_02_ describes recombination in junction dominates. It can be observed that the *J*_02_ values of the device decrease significantly as SiO_x_ is employed as a passivation layer on the front side.

### 2.3 Device Performance and Outlook of Novel Hybrid Solar Cells

**Figure 4:**
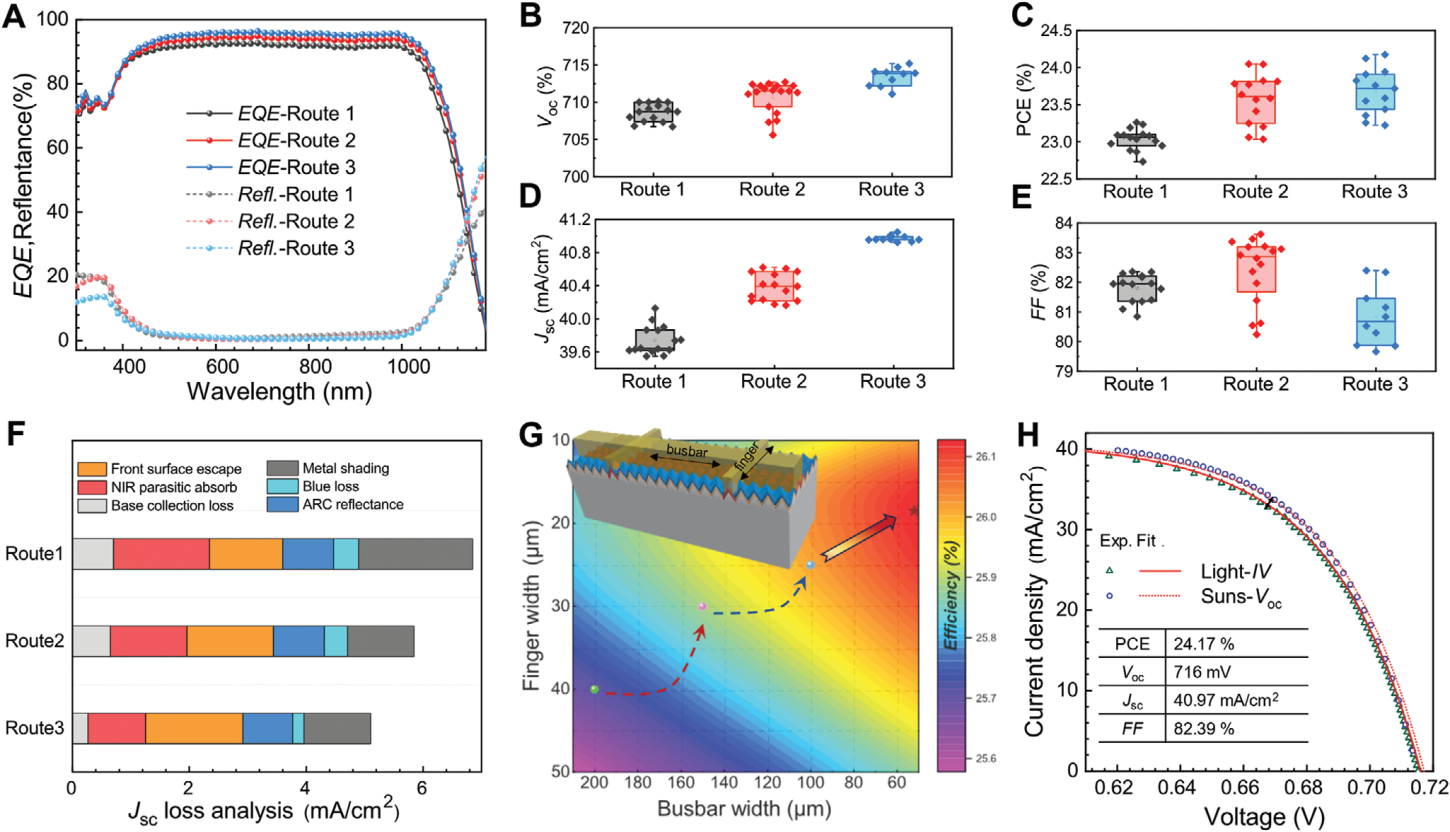
A) *EQE* and reflectance curves of hybrid solar cells with different routes. The B) *V*_OC_, C) *PCE*, D) *J*_SC_, and E) *FF* of hybrid solar cells with different routes. F) *J*_SC_ loss analysis of hybrid solar cells calculated by EQE and reflectance data. G) The theoretical efficiency of hybrid solar cells with different busbar and finger width simulated by software. H) Experimental and fit curves in Light‐*IV* and *Suns*‐*V*_OC_ state.

The amorphous silicon and ITO are deposited using the same process parameters in the fabrication. The *EQE* spectra of different routes are shown in **Figure** **4**A. SiO_x_ /SiN_x_ stack and single‐sided etching both effectively improve the passivation quality on the front side, which is reflected in the current gain across the whole wavelength. We simulate the *J‐V* and *EQE* curves (Figure S3, Supporting Information) of this device with different front surface recombination velocity (*S*_front_) and find that the trends are similar to experimental results.^[^ ^45^ ^]^ It means that electron‐hole pairs generated on the front side are more easily to recombine in BJ solar cells, which places high demands on the passivation quality of the front side. In Figure 4F, the *J*_SC_ loss analysis of three routes is compared in aspects of near infrared (NIR) parasitic absorption, ARC reflectance, metal shading, and blue loss. The improvement of *J*_SC_ in route 3 mainly benefits from the reduction of NIR absorption and metal shading. The former improvement is due to the replacement of SiO_x_ (n+) with the SiO_x_ and the successive reduction of n++ area in pattern. Corresponding to the laser pattern, our copper plating process has been adapted such as plating current and time to reduce the loss of metal shading.

The *V*_OC_ and *PCE* results of these devices with different routes are shown in Figure 4B,C, respectively. As we can see, the *V*_OC_ of solar cell is raised by ≈5 mV with the optimization of front passivation, which is consistent with the results in Figure 3A. Anomalies in TCO carrier concentration lead to reduced FF (as shown in Figure 4E) in route 3, which is mainly from the increase of *R*_s_. The gain in *J*_SC_ and *V*_OC_ increase the efficiency from 23% to 24%. Furthermore, the simulation results of device *PCE* with different width of busbar and finger are shown in Figure 4G, and the copper grid width also changes with the n++ width of busbar and finger. As we can see, the efficiency of the solar cell gradually increases as the busbar width decreases, which is due to the less surface recombination loss of dopant and contact area. Considering the trade‐off between passivation and contact performance, the efficiency of solar cell reaches the highest when the finger width is 17–19 µm. The corresponding simulated performance of *FF*, *J*_SC_, and *V*_OC_ are shown in Figure S4 (Supporting Information). It is worth noting that our simulation does not take into account the additional optical and recombination losses of pad points used to contact for conducting current (as shown in Figure S4A, Supporting Information) in the experimental plating process, so the performance parameters from simulated results is much higher than the experimental value. In future mass production, the pad points can be removed using light‐induced electroplating equipment and the efficiency achieved will be close to our simulation results. Figure 4H exhibits the experimental measurement results of light *I‐V* and *Suns*‐ *V*_OC_ curves, while the fitted data are indicated by red lines. The efficiency of champion device achieves 24.17% with a high *V*_OC_ of 716 mV. The *R*_s_ loss is reflected in the gap between the *I‐V* and *Suns*‐ *V*_OC_ curves, and this anomaly that can be attributed to fluctuations in the water vapor content during the manufacturing process of the TCO film.

**Figure 5:**
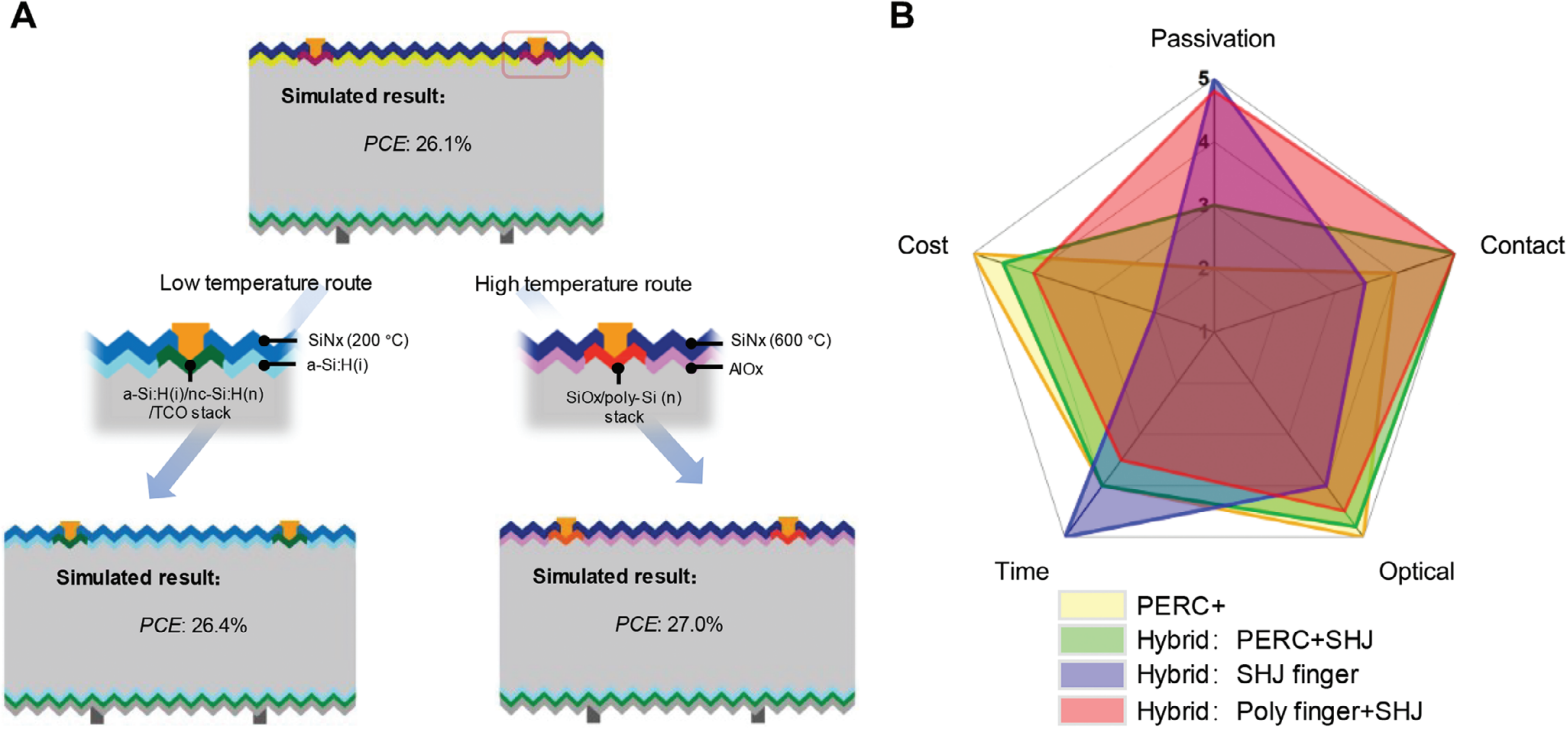
A) Structure diagram and simulation results of different hybrid solar cells. B) scores of hybrid solar cells in terms of performance and fabrication.

Although PERC and heterojunction hybrid cells can achieve conversion efficiencies of over 26%, this is still not the upper limit for such structures. As shown in **Figure** **5**A, we propose two candidate transport layers instead of the n++ structure mentioned above. In *a*‐Si:H(i)/*nc*‐Si:H(n)/TCO stack, a low‐temperature (<200 °C) deposition process of *a*‐Si:H(i)/SiN_x_ passivation layer is uesd on the front side. In another route, the diffusion temperature of SiO_x_/poly‐Si(n) stack exceeds 900 °C, employing the high‐temperature passivation of AlO_x_/SiN_x_ layers. We provide scores for each aspect of the solar cells mentioned in the paper through the radar chart shown in Figure 5B, including: passivation, contact, optics, cost and time. The hybrid of *nc*‐Si:H(n) finger and *nc*‐Si:H(p) has the highest score of passivation, and the *J*_0_ of amorphous silicon is lower than 1fA/cm^2^, giving the hybrid solar cell a higher *V*_OC_ upper limit. Extreme passivation brings about a drop in contact scores, and the contact and square resistance of microcrystalline silicon raises cell *R*_s_ and lowers the upper limit of *FF*. The front localized transmission structures place high demands on contact resistance, making poly finger structure a good choice. Even though the phosphorus diffusion leads to extra Auger recombination in silicon bulk, the insertion of poly‐Si(n) and SiO_x_ effectively avoided direct contact between the metal and the silicon surface. Parasitic absorption in the finger structure is the main reason for the reduced optical scores of the hybrid cells, but the localized structure still gives them an advantage over heterojunctions in terms of optics. Expensive low‐temperature PECVD equipment for amorphous silicon deposition is the most frequently used in SHJ finger structures. In terms of process time, the low‐temperature film deposition rate for heterojunctions is fast. The diffusion of phosphorus in poly or n++ requires a longer process time, in addition to the hydrogenation annealing in high‐temperature processes which usually takes 1–2 hours.

## 3 Conclusion

In summary, we propose a hybrid route for silicon solar cells including phosphorus diffusion and amorphous silicon deposition. The selectivity of the n++ structure without FSF is evaluated on the basis of the passivation and contact properties. Furthermore, systematic power loss simulations based on the free energy loss analysis (FELA) have been performed for traditional PERC and hybrid solar cells. The less metal shading and good contact characteristics of plating copper grid are demonstrated. In terms of the overall process, we propose three routes for optimization of front‐side passivation. The SiO_x_/SiN_x_ stack is employed instead of SiO_x_(n+) to avoid leakage at the inverted pyramid edge and the single‐side etching process is used to protect front passivation, resulting in an increase in *V*_OC_ of >5 mV. We measure the *EQE* and analyze the *J*_SC_ loss to calculate the optical improvement for different routes. The trend of *J*_SC_ with busbar and finger width is simulated to predict the potential of plating process. The champion device achieves an efficiency of 24.17% and a high *V*_OC_ of 716 mV. Simulated results indicate that hybrid solar cells with PERC and SHJ can achieve efficiencies above 26.1%. We have also predicted several hybrid architectures including amorphous silicon and poly silicon finger with simulated efficiencies of 26.4% and 27.0%, respectively. This study presents several test methods and simulation for hybrid solar cells. Furthermore, according to comprehensive scores such as passivation contact and cost, we provide an objective evaluation of the performance commercialization potential of hybrid solar cells.

## 4 Experimental Section

The device models with different passivation and contact structure are built by simulation software Quokka 3, which numerically solves the 2D/3D steady‐state charge carrier transport in silicon‐based devices with satisfying‐precision. The free energy loss analysis implemented in simulation is comprehensively described in Brendel et al.

Czochralski c‐Si wafers were produced by LONGi Company with an initial thickness of 170 µm and the electrical resistivity of 1.1–1.5 Ω·cm. The wafers were firstly etched in 20 vol% alkaline solution at 75°C for 2 min to polish the saw damage, followed by formation of surface pyramids via immersion in 2.1 vol.% alkali solution at 80 °C for 8 min. Subsequently, they underwent standard RCA cleaning to remove surface complex organics and metal ions. Before use, these wafers sustained there‐wash process for 2 min in 2.0% hydrofluoric acid water solution to remove the surface oxide. The *a*‐Si:H(i) and *nc*‐Si:H(p), with thicknesses of 7 and 20 nm respectively, were deposited on the backside of dipped wafers utilizing very‐high‐frequency plasma‐enhanced chemical vapor deposition (RF‐PECVD) technology, specifically the Ideal Energy Sunflower system operating at 40.68 MHz, at a temperature of 471 K. The silicon nitride (SiN_x_) films applied to the front side of the ideal device were fabricated and controlled via PECVD technology sourced from Shenzhen S.C. New Energy Technology Corporation, at a temperature of 673 K. Low‐pressure chemical vapor deposition (LPCVD), which employs heat energy to activate substances and promote their thermal decomposition or chemical reactions, was utilized to facilitate the diffusion of phosphorus (P) and the deposition of SiO_x_ (n+) or SiO_x_ films.

In the preparation process of hybrid solar cells, phosphorus diffusion was carried out on the surface of the textured silicon wafers. SiN_x_ film deposited on the front side as a mask. A 532 nm picosecond laser was used to remove the area without selective transport. Next, the second texture was performed to form the new pyramids, and the passivation layers were deposited on the front (SiO_x_/SiN_x_) and back (*a*‐Si:H(i)/*nc*‐Si:H(p)/TCO) surfaces separately. Then, a 355 nm picosecond laser was performed to open the contact area on the front to plate Cu grid. Finally, the rear Ag grid was formed by screen printing. To ensure the formation of a copper grid line with a thickness of ≈20 µm at the laser‐opened area, a SiN_x_ layer on the front side serves as a barrier during the plating process. Initially, a nickel seed layer is deposited to facilitate low contact resistance with the silicon substrate. Then, a copper grid is formed, characterized by its low line resistance. Finally, the copper grid is encapsulated with a nickel layer to safeguard against oxidation (as shown in Figure S1C,D, Supporting Information). In Figure 1d, Region 1 employs SiN_x_ as a masking layer to delineate an area slightly larger than the spot designated for testing the *EQE* of the P‐diff/SiN_x_ stack. The sample under investigation adheres to the specified cell architecture.

## Conflict of Interest

The authors declare no conflict of interest

## Supporting information



Supporting Information

## Data Availability

Data sharing is not applicable to this article as no new data were created or analyzed in this study.
