# Supplementary material for: Optimization Strategies and Efficiency Prediction for Silicon Solar Cells with Hybrid Route of PERC and SHJ Passivation Contact
Source: Adv Sci (Weinh). 2025 Feb 24;12(15):2411965. doi: 10.1002/advs.202411965 (PMC12005823; doi:10.1002/advs.202411965)
Supplement: Supplementary file 1 — Supporting Information [file ADVS-12-2411965-s001.docx]

**Supplementary Material**

**Optimization Strategies for Silicon Solar Cells with Hybrid Route of PERC and SHJ Passivation Contact to Achieve more than 24% Efficiency**

Zixiao Zhou^1,3,4^, Qian Kang^1,2*^, Zhaoqing Sun^1,3,4^, Yongcai He^1,3,4^, Jingjie Li^1^, Lu Wu^3,4^, Chang Sun^3,4^, Chaowei Xue^3,4^, Minghao Qu^3,4^, Zilong Zheng^1^, Bo Wang^1^, Hui Yan^1^, Xixiang Xu^3,4^, Yongzhe Zhang^1,2*^

^1^ College of Materials Science and Engineering, Key Laboratory of New Functional Materials of Ministry of Education, Beijing University of Technology, Beijing 100124, China

^2^ School of Information Science and Technology, Key Laboratory Optoelectronics Technology of Ministry of Education, Beijing University of Technology, Beijing 100124, China

^3^ LONGi Green Energy Technology Co., Ltd, Xi’an, 710016, China

^4^ LONGi Central R&D Institute, Xi’an, 712000, China

Corresponding author at:

College of Materials Science and Engineering, Key Laboratory of New Functional Materials of Ministry of Education, Beijing University of Technology, Beijing 100124, China

School of Information Science and Technology, Key Laboratory Optoelectronics Technology of Ministry of Education, Beijing University of Technology, Beijing 100124, China

Corresponding authors:

E-mail addresses: (Q. Kang), kangqian@[bjut.edu.cn](mailto:bjut.edu.cn) (Y.Z Zhang), yzzhang[@bjut.edu.cn](mailto:qiankang@bjut.edu.cn)

**Table S1.** Detailed input parameters of simulation in Figure 1C and 4G

|  | PERC+^[1-3]^ | Hybrid^[3-5]^ |
| --- | --- | --- |
| Wafer thickness | 170 μm | 170 μm |
| Wafer resistivity | 1.0 Ω·cm | 1.0 Ω·cm |
| Front grid width | 30 μm | 20 μm |
| Front passivation *J*_0_ | 12 fA/cm^2^ | 4 fA/cm^2^ |
| Front contact *J*_0_ | 1400 fA/cm^2^ | 150 fA/cm^2^ |
| Front contact *ρ*_c_ | 1 mΩ·cm^2^ | 0.73 mΩ·cm^2^ |
| Rear grid width | 30 μm | 40 μm |
| Rear passivation *J*_0_ | 1 fA/cm^2^ | 0.5 fA/cm^2^ |
| Rear contact *J*_0_ | 400 fA/cm^2^ | 2 fA/cm^2^ |
| Rear contact *ρ*_c_ | 5 mΩ·cm^2^ | 0.4 mΩ·cm^2^ |
| Rear TCO *R*_sheet_ | / | 40 Ω/sq |


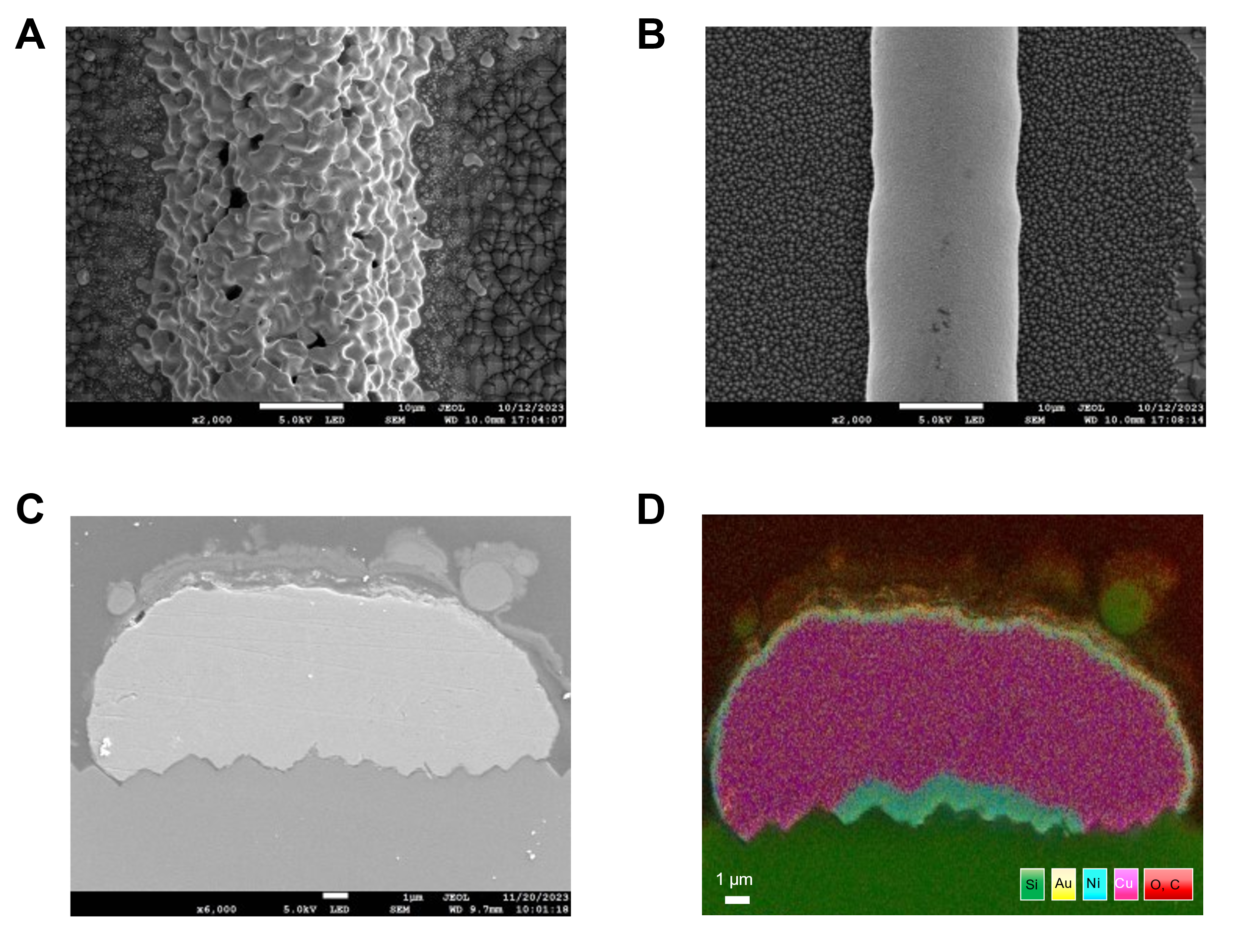


**Fig S1.** Surface morphology view of (A) high-temperature screen printing and (B) copper planting; (C) cross-sectional view and (D) EDS analysis of copper grid.


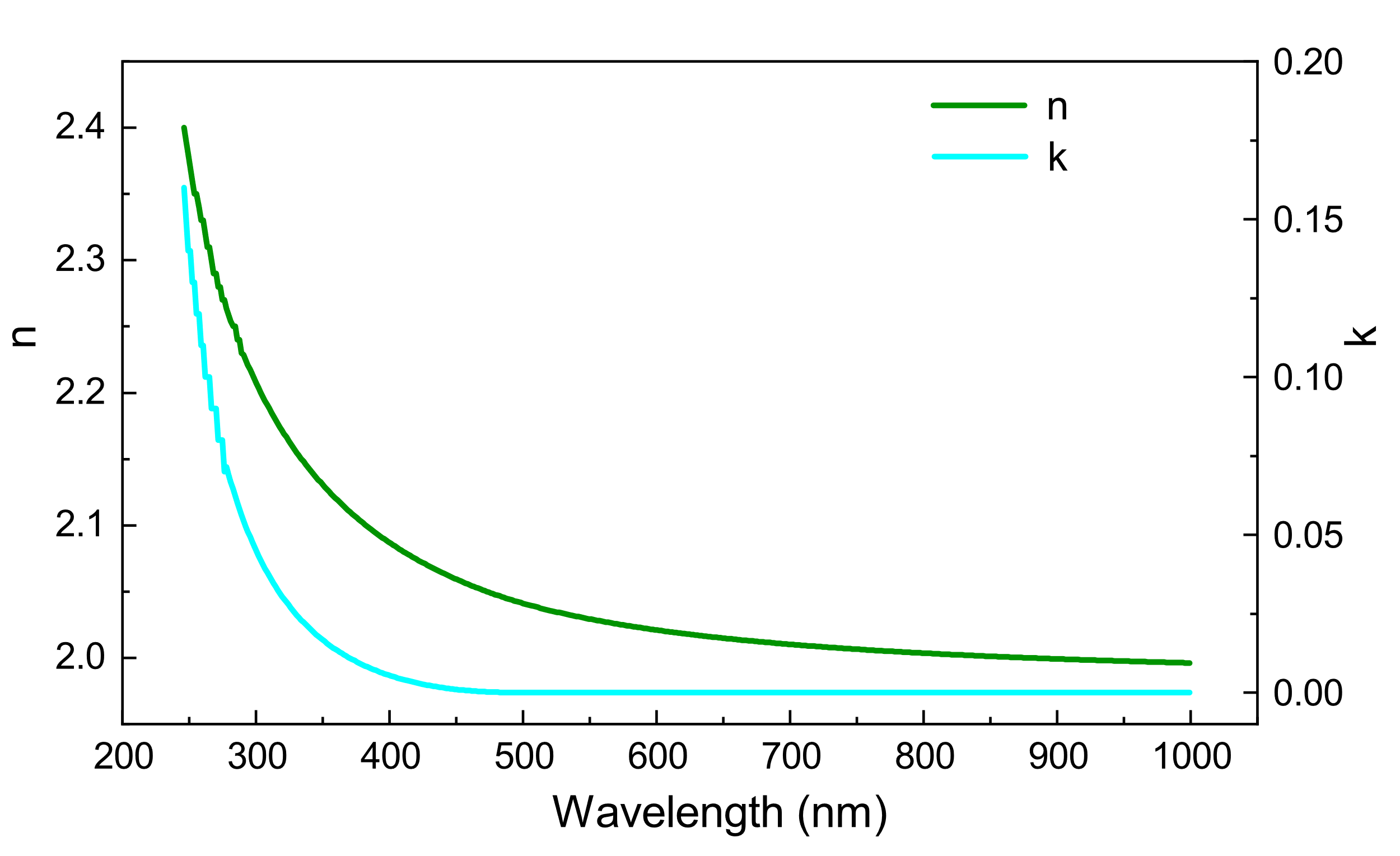


**Fig S2.** *n* and *k* value of SiN_x_ films with the change of wavelength.


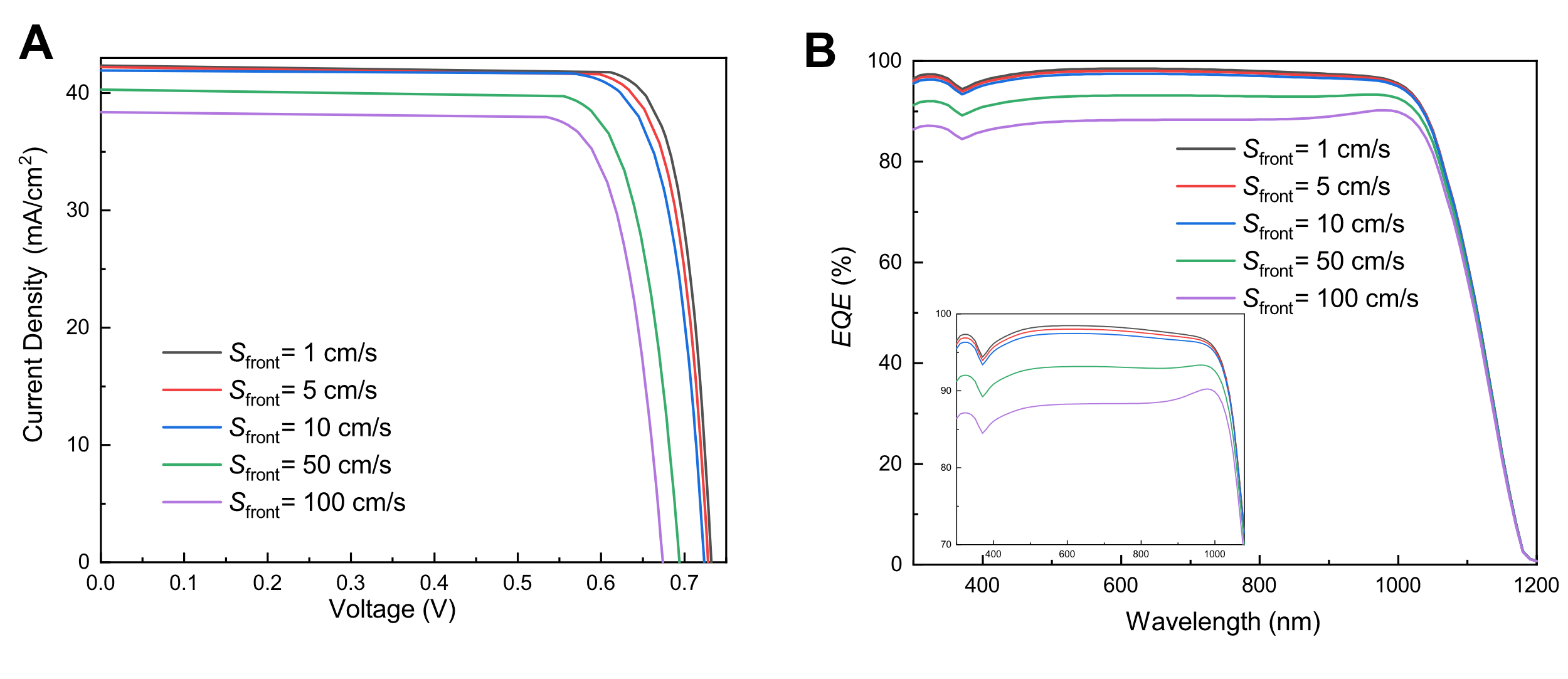


**Fig S3.** Simulation results of (A) *J-V* and (B) *EQE* curves with different front surface recombination velocity of hybrid soalr cell model.^[6]^


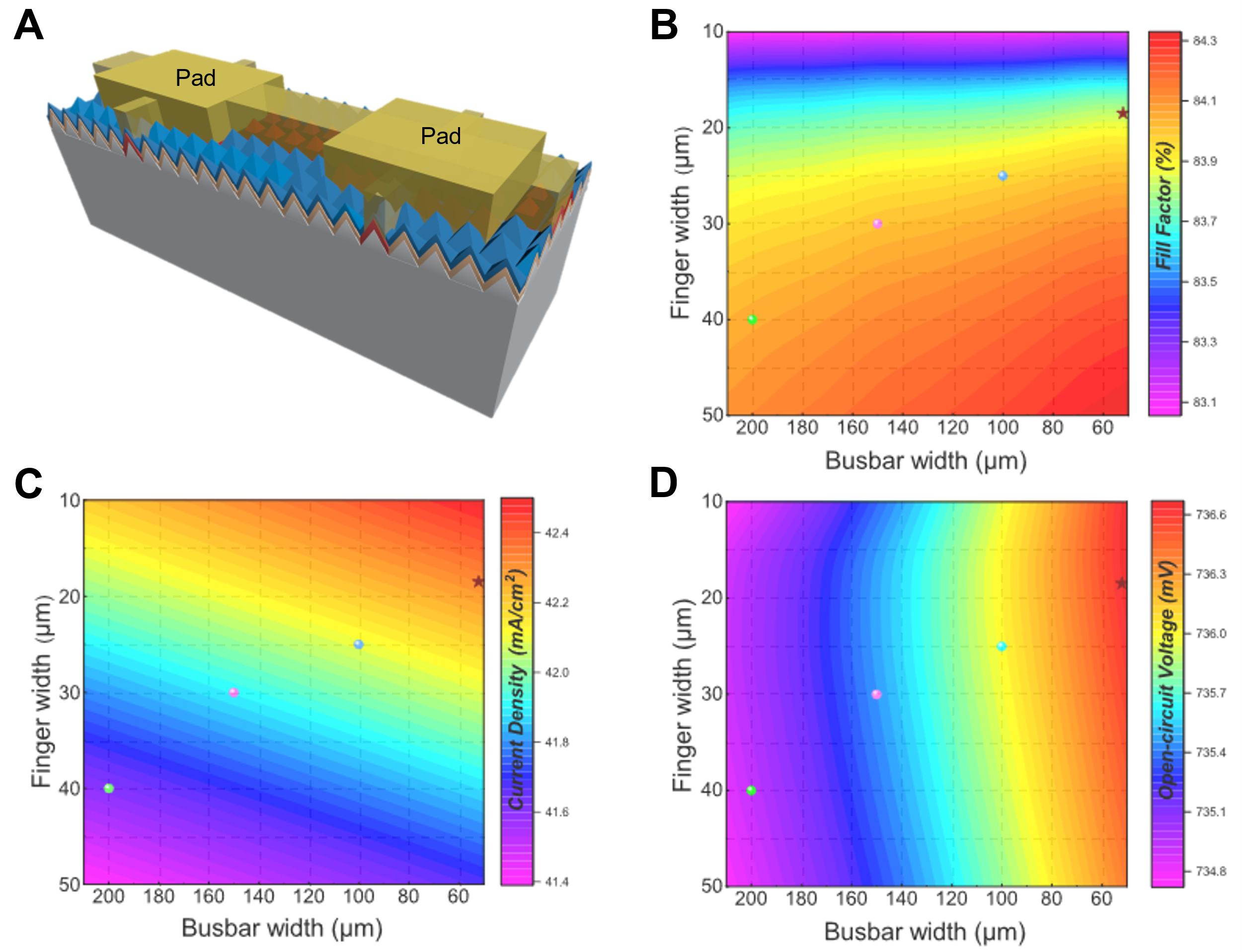


**Fig S4.** (A) Electroplating pad point diagram; simulated (B) *FF* (C) *J*_SC_ and (D) *V*_OC_ of hybrid solar cells with different finger and busbar width.

**Referance**

[1] T. Dullweber, *Sol. Energy Mater. Sol. Cells* **2020,** 212, 110586.

[2] J. Schmidt, R. Peibst, R. Brendel, *Sol. Energy Mater. Sol. Cells* **2018**, *187*, 39.

[3] R. Brendel, R. Peibst, *IEEE J. Photovolt.* **2016**, *6*, 1413.

[4] W. Long, S. Yin, F. Peng, M. Yang, L. Fang, X. Ru, M. Qu, H. Lin, X. Xu, *Sol. Energy Mater. Sol. Cells* **2021**, *231*, 111291.

[5] H. Lin, M. Yang, X. Ru, G. Wang, S. Yin, F. Peng, C. Hong, M. Qu, J. Lu, L. Fang, C. Han, P. Procel, O. Isabella, P. Gao, Z. Li, X. Xu, *Nat. Energy* **2023**, *8*, 789.

[6] Q. Yang, K. Bittkau, A. Eberst, U. Rau, K. Ding, *Sol. Energy Mater. Sol. Cells* **2024**, *273*, 112953.
